# The diagnostic and therapeutic challenge of atrial flutter in children: a case report

**DOI:** 10.1186/s13052-023-01542-4

**Published:** 2023-10-09

**Authors:** Angelica De Nigris, Mattia Arenella, Giangiacomo Di Nardo, Giovanni Maria Di Marco, Annunziata Mormile, Daria Lauretta, Caterina De Simone, Angela Pepe, Rosaria Cosimi, Rossella Vastarella, Antonietta Giannattasio, Giovanni Salomone, Silverio Perrotta, Speranza Cioffi, Pierluigi Marzuillo, Vincenzo Tipo, Luigi Martemucci

**Affiliations:** 1 Department of Woman, Child and General and Specialized Surgery, University of Campania “Luigi Vanvitelli”, Via Luigi De Crecchio, Naples, 80138 Italy; 2 Division of Cardiology, Department of Pediatrics, Santobono-Pausilipon Children Medical Hospital, Naples, 80129 Italy; 3 Department of Translational Medical Science, Section of Pediatrics, University of Naples “Federico II”, Naples, 80126 Italy; 4 Department of Medicine, Surgery and Dentistry “Scuola Medica Salernitana”, Pediatrics Section, University of Salerno, Baronissi, 84081 Italy; 5 Pediatric Emergency and Short Stay Unit, Santobono-Pausilipon Children’s Hospital, Naples, 80129 Italy; 6 Pediatric Gastroenterology Unit, Santobono-Pausilipon Children’s Hospital, Naples, Italy

**Keywords:** Atrial flutter, Children, Dysrhythmia, Left upper atrial rhythm, SARS-CoV-2

## Abstract

**Background:**

Palpitations represent a common cause for consultation in the pediatric Emergency Department (ED). Unlike adults, palpitations in children are less frequently dependent from the heart, recognizing other causes.

**Case presentation:**

A 11-year-old male came to our pediatric ED for epigastric pain, vomiting and palpitations. During the previous 6 month the patient was affected by SARS-CoV-2 (Severe Acute Respiratory Syndrome Coronavirus). Electrocardiogram (ECG) revealed supraventricular tachycardia. Therefore, adenosine was administered unsuccessfully. The administration of adenosine, however, allowed us to make diagnosis of atypical atrial flutter. Multiple attempts at both electrical cardioversion, transesophageal atrial overdrive, and drug monotherapy were unsuccessful in our patient. Consequently, a triple therapy with amiodarone, flecainide, and beta-blocker was gradually designed to control the arrhythmic pattern with the restoration of a left upper atrial rhythm. There was not any evidence of sinus rhythm in the patient clinical history.

**Conclusions:**

The present study underlines the rarity of this type of dysrhythmia in childhood and the difficulties in diagnosis and management, above all in a patient who has never showed sinus rhythm. Raising awareness of all available treatment options is essential for a better management of dysrhythmia in children

## Background

Palpitations represent a common cause for consultation in the pediatric Emergency Department (ED). Unlike adults, palpitations in children are less frequently dependent from the heart, recognizing other causes (anxiety, fear, exercise or fever). The overall incidence of dysrhythmias is 55.1 per 100,000 pediatric ED visits [1]. Nevertheless, the pediatric ED physician should be able to determine whether child requires immediate examination and referral to a cardiologist [2, 3]. Sacchetti et al. [4] conducted a retrospective review of cardiac dysrhythmias in children presenting to the ED, and revealed that sinus tachycardia was the most commonly reported dysrhythmia (50%), followed by supraventricular tachycardia (13%), nonspecific dysrhythmias (10.6%), bradycardia (6%), and atrial fibrillation (4.6%).

Here we report the case of a 11-year-old boy with structurally normal heart and a history of previous SARS-CoV-2 (Severe Acute Respiratory Syndrome Coronavirus 2) infection, admitted to the pediatric ED for tachycardia determined by a rare cause. Furthermore, it is emphasized that lack of evidence-based therapies makes management of dysrhythmias particularly difficult and complex in childhood.

## Case presentation

A 11-year-old male presented to the pediatric ED with epigastric pain, vomiting and palpitations.

Patient’s medical history included a pauci-symptomatic SARS-CoV-2 infection in the previous six months, confirmed by the finding of Immunoglobulin (Ig) G for SARS-CoV-2 in the absence of IgM, and was negative for syncope or other cardiac manifestations. There was no familiarity for congenital heart disease or sudden cardiac death.

At admission general conditions were not so poor, with body temperature of 36.5 °C, oxygen saturation in room air of 99%, respiratory rate of 15 breaths/min, systemic blood pressure of 95/65 mmHg, except for a heart rate (HR) of 250 beats/min. The anthropometric parameters were: weight 32 kg, height 135 cm, body surface area 1.095 m^2^.

Electrocardiogram (ECG) revealed supraventricular tachycardia with a narrow QRS; atrial rate (AR) was equal to ventricular (VR) and was 230 b/min. Echocardiography revealed mild depressed biventricular function with ejection fraction (EF) of 53%, left atrial dilatation and moderate mitral regurgitation.

Adenosine was administered twice: first dose 0,1 mg/kg; second dose 0,2 mg/kg - both ineffective.

**Fig. 1:**
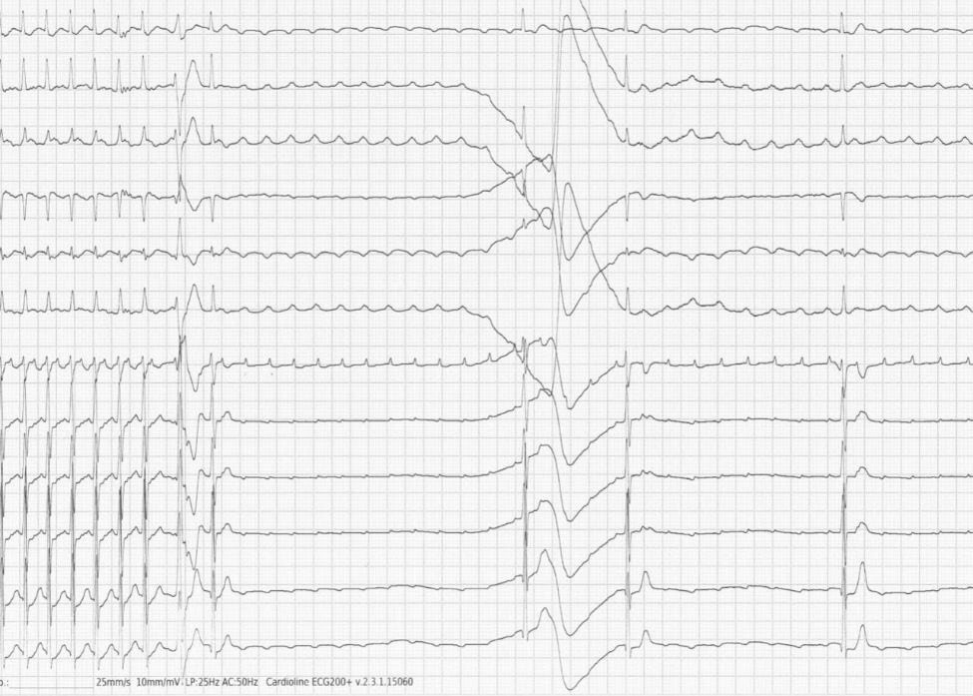
Atypical atrial flutter. The electrocardiogram shows the atypical atrial flutter (AFL) revealed after the administration of adenosine

The administration of adenosine (0,1 mg/kg), however, allowed us to make diagnosis of atypical atrial flutter (AFL) (Fig. 1).

Therefore, therapy with intravenous flecainide (2 mg/kg in 15 min) was attempted with no response.

Continuous intravenous amiodarone (300 mg/m^2^/d) was started leading to unsatisfactory heart rate control and, above all, unsuccessful rhythm control.

Due to the persistence of AFL, synchronized direct current cardioversion (first shock at 75 J and second one at 100 J) was performed without success.

Therefore, oral propranolol (1 mg/kg/d) was added to therapy with a better heart rate control but not as expected. After 24 h intravenous amiodarone was discontinued and oral amiodaron (150 mg/m^2^/d) was started.

In the following days, further attempts at both synchronized direct current cardioversion and transesophageal atrial pacing were ineffective to stop dysrhythmia.

Therefore, propranolol posology was increased to 1.5 mg/kg/d and oral flecainide (4.7 mg/d) was introduced. The result was represented by a gradual reduction of ventricular response and, after two days of this therapy, ECG demonstrated the dysrhythmia disorganization with the appearance of polyfocal atrial tachycardia.

Due to the persistence of protracted phases of ectopic atrial tachycardia, flecainide was further increased to 5 mg/d with progressive reduction of the arrhythmic pattern.

During the hospitalization the patient was monitored using continuous ECG, several Holter ECG and 12-lead ECG more than once a day, as well as echocardiographic evaluation and periodical biochemistry (the latter always within normal range).

**Fig. 2:**
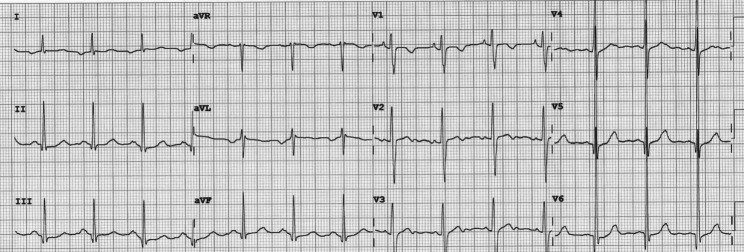
Basal electrocardiogram pattern. The electrocardiogram shows the basal pattern of the patient characterized by a stable upper left atrial rhythm

**Fig. 3:**
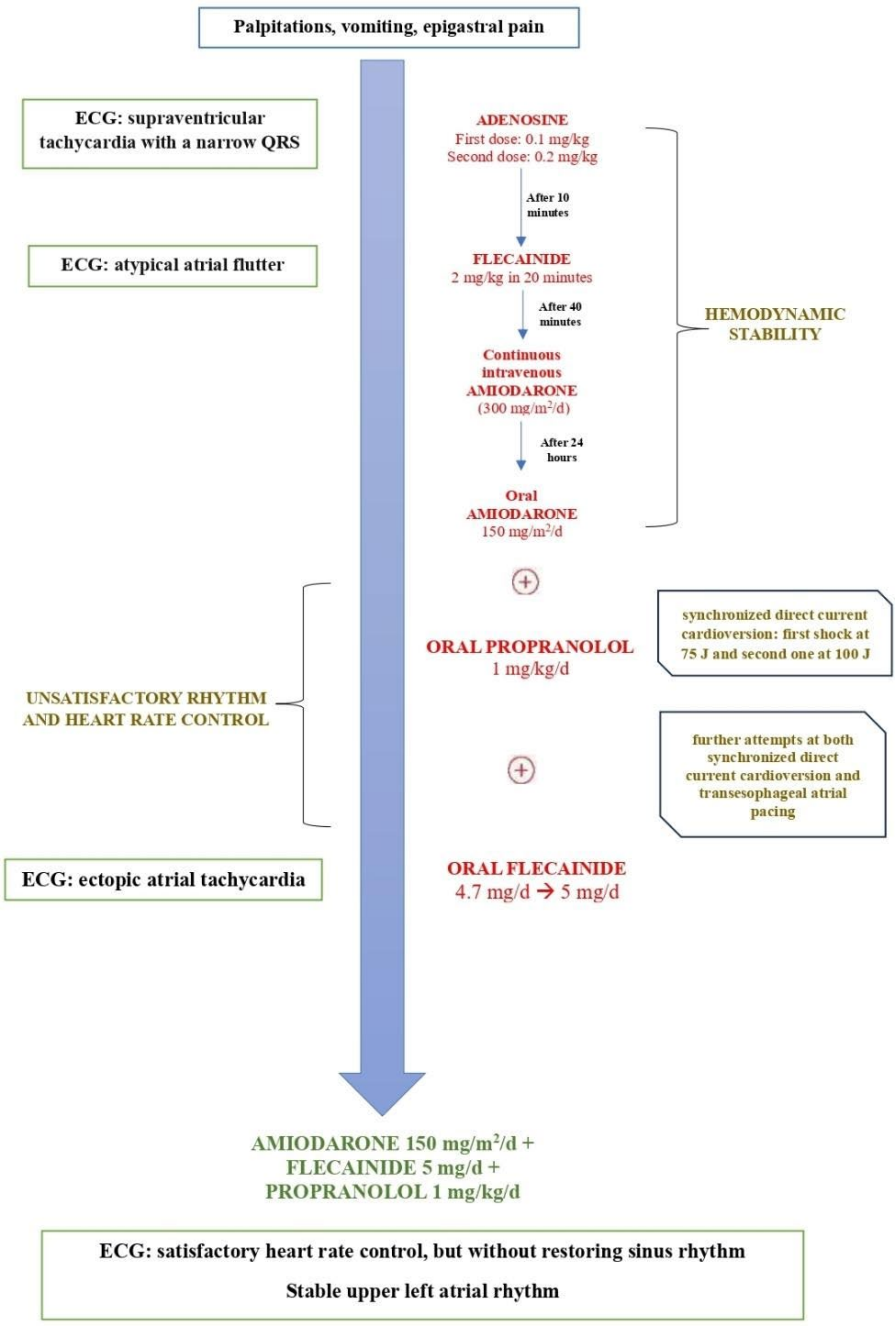
Therapeutic management step by step

Combined therapy with amiodarone (150 mg/m^2^/d), propranolol (1.5 mg/kg/d) and flecainide (5 mg/Kg/d) was able to ensure satisfactory heart rate control, but without restoring sinus rhythm and appearance of stable upper left atrial rhythm with HR of 85 beats/min (Figs. 2 and 3).

Echocardiography also showed normalization of ventricular function and atrial size. Mild mitral regurgitation persisted due to kneeling of the anterior valve leaflet.

In consideration of evident clinical and echocardiographic improvement, the patient was discharged and underwent follow-up.

After one month of follow-up, to date, this therapy continues to show satisfactory results in terms of clinical conditions and control of dysrhythmia.

As further confirmation, we asked the parents for a previous ECG of the child and we learned that he already had a baseline left upper atrial rhythm two months before the arrhythmic event.

Subsequent diagnostic investigations, such as cardiac magnetic resonance and endomyocardial biopsy, may be useful to define the nature of this rare case of adolescent atrial flutter and its future therapeutic management.

## Discussion and conclusions

Atrial flutter (AFL) represents a rare dysrhythmia in children; it is characterized by fast and regular atrial activity (240–500 beats per minute) usually caused by macro-reentry circuit within the atrial wall [5]. It is frequently associated with 2:1 atrioventricular (AV) conduction. Vagal stimulation, adenosine, or beta blockers can increase the degree of AV block and unmask atrial waves on the ECG that are often enough for diagnosis [5–9].

AFL is divided, also in childhood, into two categories: typical and atypical forms [10].

Atypical atrial flutter includes a broad spectrum of other macro-reentrant tachycardias in which the wave front does not travel around the tricuspid annulus [11].

In our case, an atypical AFL was diagnosed with probable origin in the left atrium [11].

AFL without any underlying myocardial disease (“lone AFL”) is uncommon in childhood and most frequent in the neonatal period [5, 6].

Beyond this period, it is mainly found in children with congenital heart defects, especially after cardiac surgery performed within atria, or in case of systemic disease with immaturity of the conduction system of the heart [6, 7].

In a multicenter study including 380 patients with atrial flutter aged 1 to 25 years, only 8% had a structurally normal heart [7].

This low incidence precludes large pediatric studies of these dysrhythmias with a lack of evidence-based recommendations for management, especially in children and adolescents.

Recommended treatment to restore neonatal sinus rhythm includes electrical cardioversion, transesophageal atrial pacing, or antiarrhythmic drugs [6].

Synchronized electrical cardioversion or transesophageal atrial overdrive is the first choice of therapy for both a stable and unstable infant [6, 12, 13].

In children and adolescents, depending on the clinical presentation and antiarrhythmic drug efficacy, additional treatments are electrophysiological study and catheter ablation of the AFL substrate [14].

The recommended drugs are digoxin with the addition of flecainide or amiodarone in case of therapeutic failure. Although they take some time to restore sinus rhythm, they may be tried in a stable patient [6].

Digoxin has been widely used as a positive inotropic agent in patients with systolic heart failure and as a negative chronotropic agent in patients with AFL [15].

In our case, it was previously avoided because its use might be potentially dangerous during electrical cardioversion [16]. Multiple attempts at both electrical cardioversion, transesophageal atrial overdrive, and drug monotherapy were unsuccessful in our patient. Consequently, a triple therapy with amiodarone, flecainide, and beta-blocker was gradually and empirically designed to control the arrhythmic pattern. In our center, we prefer to exploit the synergistic effect of drugs with different mechanisms of action rather than increasing the dosages of single drugs up to the upper limits of the therapeutic range.

In addition to the clinical and echocardiographic improvement, the efficacy was further confirmed by the restoration of the basal rhythm, pre-existing to the arrhythmic event, even if not originating from the sinus node.

Ectopic atrial rhythm (EAR) could be a normal characteristic in healthy children [17]; this pattern could also be associated with cardiac tissue alterations due to surgery (like cardiopathies) or myocardial changes (like Fabry’s disease or left ventricular noncompaction). In our case there was no evidence of tissue damage due to surgical fibrosis or myocardial abnormalities. Otherwise, the patient was affected by Sars-CoV-2 during the previous 6 months and his ECGs over this period always showed stable left upper atrial rhythm; there was no availability of previous ECGs prior to the infection. Common cardiac manifestations in SARS-CoV-2 infection and Multisystem Inflammatory Syndrome in Children are ventricular disfunction, coronary anomalies and a wide spectrum of rhythm abnormalities [2].

In this clinical case, SARS-CoV-2 infection might be the trigger for patient’s persistent EAR as well as for the atrial atypical flutter episode.

Data on “lone AFL”, i.e. in the absence of any underlying myocardial disease as in our case, are limited [12, 18].

It is now known that atrial flutter most often occurs as a single episode without late recurrence. Therefore, long-term prophylaxis is not routinely recommended [6].

In case of recurrent episodes, especially in older children, ablation should be considered both for the low procedural risks and for high efficacy as an alternative to drugs [6, 14, 19].

Recently, the role of endomyocardial biopsy in revealing underlying cardiac disease, has been highlighted in a significant number of patients with presumed “lone AFL”.

Furthermore, the use of cardiac magnetic resonance and possible genetic tests is recommended in order to set a disease-specific treatment [20].

For this reason, these investigations represent the next steps in our patient’s diagnostic work-up.

Both the results of the diagnostic investigations and the response to drug therapy will guide us in the future therapeutic management of the patient.

The present study underlines the rarity of this type of dysrhythmia in childhood and the difficulty of its diagnosis and management. F waves may be difficult to see initially on ECG due to accelerated ventricular response: in our case, what at first appeared to be supraventricular tachycardia, after the use of adenosine that slowed atrial frequency, turned out to be an atrial flutter.

Therefore, raising awareness of all available treatment options is essential for better management of dysrhythmia in children.

Further studies on larger group of patients beyond the neonatal period are essential to establish the most appropriate and effective combinations of drugs to recommend in the pediatric age.

## Data Availability

All data generated or analyzed during this study are included in this published article.

## Abbreviations

AFL: Atypical atrial flutter
AR: Atrial rate
AV: Atrioventricular
EAR: Ectopic Atrial Rhythm
ECG: Electrocardiogram
ED: Emergency Department
EF: Ejection Fraction
HR: Heart rate
Ig: Immunoglobulin
SARS-CoV-2: Severe Acute Respiratory Syndrome Coronavirus 2
VR: Ventricular rate
